# Genetic Characterization and Molecular Docking of Flavonoid Compounds Against *Klebsiella pneumoniae* and Bovine Herpesvirus Type 1 (BoHV‐1) Isolated From Clinical Dairy Cattle Cases

**DOI:** 10.1155/ijm/8888183

**Published:** 2026-06-10

**Authors:** Karrar Jasim Hamzah, Noor R. Abady, Mohammed Zuhair Naji, Shamsaldeen Ibrahim Saeed, Nor Fadhilah Kamaruzzaman, Mohammed Dauda Goni

**Affiliations:** ^1^ Department of Veterinary Internal and Prevention Medicine, College of Veterinary Medicine, Al-Qasim Green University, Babylon, Iraq, uoqasim.edu.iq; ^2^ Department of Veterinary Microbiology, College of Veterinary Medicine, Al-Qasim Green University, Babylon, Iraq, uoqasim.edu.iq; ^3^ Department of Medical Biotechnology, College of Sciences, Al-Mustaqbal University, Babylon, Iraq, mustaqbal-college.edu.iq; ^4^ Faculty of Veterinary Science, University of Nyala, Nyala, Sudan, nyalau.edu.sd; ^5^ Zoonotic and Transboundary Animal Disease Research Centre, Faculty of Veterinary Medicine, Universiti Malaysia Kelantan, Kota Bharu, Kelantan, Malaysia, umk.edu.my; ^6^ Department of Clinical Studies, Faculty of Veterinary Medicine, Universiti Malaysia Kelantan, Kota Bharu, Kelantan, Malaysia, umk.edu.my

**Keywords:** antimicrobial resistance, BoHV-1, flavonoids, *Klebsiella pneumoniae*, molecular docking

## Abstract

*Klebsiella pneumoniae* and Bovine Herpesvirus Type 1 (BoHV‐1) are two significant pathogens of dairy cattle that cause debilitating diseases in the respiratory, reproductive, and mammary gland systems. Conventional antimicrobials have become less effective due to the emergence of antimicrobial‐resistant strains, and other molecules are needed for therapeutic purposes. This study was designed to identify *Klebsiella pneumoniae* and BoHV‐1 infection in dairy cows and to assess the antimicrobial activity of flavonoids derived from plants as alternative drugs. A total of 50 samples were collected, including 40 milk samples from cows with clinical mastitis and 10 nasal swabs from cattle with respiratory symptoms between December 12, 2023, and April 15, 2024. Mastitis‐associated *Klebsiella pneumoniae* was recovered from 15/40 milk samples (37.5%), and BoHV‐1 was found in nasal swabs of 50% of the animals. The molecular docking studies found that the binding affinity of quercetin with *Klebsiella pneumoniae* OmpA (−8.2 kcal/mol) was higher than the binding affinity of cefotaxime with the same OmpA (−6.9 kcal/mol). Kaempferol proved to have dual inhibitory activity against both pathogens (−7.5 kcal/mol). All isolates contained a conserved G→T transversion at Position 21, which is based on multiple sequence alignment with the BoHV‐1 gB reference sequence MG407791.1. The results indicate that flavonoids, specifically quercetin and kaempferol, showed good binding properties with the targeted bacteria and viruses and warrant further research to investigate their therapeutic potential.

## 1. Introduction

The emergence of antimicrobial‐resistant (AMR) pathogens within livestock production systems represents a growing threat to both veterinary and public health globally [1]. Among them, AMR in *Klebsiella pneumoniae* and Bovine Herpesvirus Type 1 (BoHV‐1) are important pathogens responsible for severe infections in dairy cattle, including respiratory, reproductive, and mastitis. These infections are major contributors to economic losses in the dairy industry, estimated to exceed two billion dollars annually worldwide [2, 3]. The increasing prevalence of multidrug‐resistant *Klebsiella pneumoniae* [4] and the limited efficacy of current antiviral agents against BoHV‐1 [5] underscore the urgent need for novel therapeutic approaches. The reduced effectiveness of conventional antibiotics toward *Klebsiella pneumoniae* is attributed to rapid acquisition of multiple resistance mechanisms, such as the production of diverse *β*‐lactamases and structural modifications of membrane proteins [6, 7]. Similarly, BoHV‐1 exhibited variable susceptibility to acyclovir and other nucleoside analogs, posing additional challenges for effective antiviral therapy [8]. Thus, there is growing interest in plant‐derived compounds, which have shown promising effects against *Klebsiella pneumoniae*. Flavonoids are organic compounds characterized by a range of phenolic structures that have been recognized to contain several biological properties, including antimicrobial, antioxidant, antiallergic, anti‐inflammatory, antileishmanial, and cytotoxic antitumor activity [9–12]. Flavonoids exhibit antimicrobial effects through three mechanisms: directly destroying the bacteria, synergistically enhancing antibiotics, and reducing microbial pathogenicity [13]. Notably, flavonoids have been discovered to inhibit the efflux pump of methicillin‐resistant *Staphylococcus aureus* [14] and possess inhibitory activity against various types of beta‐lactamases that inactivate beta‐lactam antibiotics. Quercetin and related polyphenols have shown promising activity against bacterial and viral pathogens through mechanisms involving enzyme inhibition, interference with nucleic acid synthesis, and disruption of microbial membrane integrity [15, 16]. Recent advances in computational biology have significantly enhanced drug discovery strategies. Molecular docking, in particular, has emerged as a cost‐effective and efficient tool for predicting ligand–target interactions and identifying potential therapeutic agents [17, 18]. In this study, we selected the outer membrane protein A (OmpA) of *Klebsiella pneumoniae* as the bacterial target due to its important structural and functional roles in Gram‐negative bacteria. OmpA is required to maintain the outer membrane integrity and stability of *Klebsiella pneumoniae* and other Enterobacteriaceae [19]. Interference with OmpA function can weaken the bacterial membrane wall, thereby increasing permeability as well as susceptibility to other antimicrobial agents. Furthermore, OmpA contributes to biofilm formation, which is a significant factor in chronic infection and AMR. Certain compounds that disrupt OmpA expression or activity have been reported to reduce biofilm formation and bacterial attachment to host cells [19]. In addition, OmpA has been associated with AMR by modulating the uptake of *β*‐lactam antibiotics through porin channels. Reduced OmpA expression has been linked to decreased permeability to carbapenems and other *β*‐lactam antibiotics in multidrug‐resistant *Klebsiella pneumoniae* isolates [6]. Recent studies have also identified OmpA as a potential target for plant‐based antimicrobial compounds, including flavonoids [9]. This study was aimed at investigating the occurrence and molecular characteristics of *Klebsiella pneumoniae* and BoHV‐1 infections in dairy cattle and at evaluating the therapeutic potential of selected plant‐derived flavonoids as an alternative antimicrobial agent.

## 2. Methods and Materials

### 2.1. Sample Collection

This work was conducted between December 12, 2023, and April 15, 2024. A total of 40 milk samples were collected aseptically from dairy cows displaying clinical signs of mastitis, including udder inflammation, milk clots, and fever. Milk samples were collected prior to antibiotic administration. At the same time, 10 nasal swab samples were collected from cattle showing respiratory symptoms (such as nasal discharge, coughing, and fever) and were not associated with clinical mastitis. All samples were placed in sterile containers and transported on ice. All experimental procedures involving animals were conducted in accordance with institutional biosafety and ethical guidelines and were approved by the Animal Ethics and Scientific Research Committee, College of Veterinary Medicine, Al‐Qasim Green University, Iraq (Approval No. QG/11/2025).

### 2.2. Bacterial Isolation and Identification

Out of 40 milk samples collected from cows exhibiting clinical mastitis, only 15 samples showing severe clinical manifestations (including marked udder inflammation, abnormal milk consistency, and systemic signs such as fever) were subjected to bacteriological isolation and molecular confirmation. We were focused on and targeting *Klebsiella pneumoniae* for molecular docking and genetic characterization rather than representing the complete etiological spectrum of mastitis within the entire sampled population. For isolation of bacteria, 100 *μ*L of milk from each sample was plated onto a MacConkey plate and incubated at 37°C aerobically for 24~48 h. The suspected *Klebsiella* spp. (pink–yellow, mucoid, lactose‐positive colonies) were subject to biochemical tests [20] and molecular confirmations by using PCR using specific primers targeting the *dnaN* locus (forward: 5 ^′^‐TGAAAATCGTGGTCGG‐3 ^′^; reverse: 5 ^′^‐TCATCGCTTCGTCGTCT‐3 ^′^). PCR conditions included an annealing temperature of 58°C and amplification of a 562 bp product.

### 2.3. Viral Detection

Nasal swabs were collected from clinically affected cattle and placed in viral transport medium. Viral DNA was extracted using a commercial kit following the manufacturer′s protocol. Detection of BoHV‐1 was performed by PCR targeting the glycoprotein B (gB) gene with specific primers (forward: 5 ^′^‐TACGACTCGTTCGCGCTCTC‐3 ^′^; reverse: 5 ^′^‐GGTACGTCTCCAAGCTGCCC‐3 ^′^) as described previously [7]. The reaction produced a 478 bp amplicon under an annealing temperature of 56°C. PCR products were subjected to partial sequencing of the gB gene for molecular characterization and mutation analysis.

### 2.4. Protein Structure Preparation and Homology Models

Since no experimentally determined crystal structures of *Klebsiella pneumoniae* OmpA and BoHV‐1 DNA polymerase are available in the Protein Data Bank (PDB), their three‐dimensional structures were generated using homology modeling with the SWISS‐MODEL server. For OmpA, *Escherichia coli* OmpA (PDB ID: 1BXW; 78% identity) was used as the template. Model quality was confirmed by a Global Model Quality Estimation (GMQE) score of 0.82, a Qualitative Model Energy Analysis (QMEAN) score of −2.15, and 95.2% of residues in favored Ramachandran regions, indicating a reliable structure for docking studies. For BoHV‐1 DNA polymerase, the selected template was Herpes Simplex Virus Type 1 (HSV‐1) DNA polymerase (PDB ID: 5IQB), representing a high‐resolution structure of a related herpesvirus polymerase. Sequence alignment between BoHV‐1 DNA polymerase (GenBank Accession: AAC42156.1) and the HSV‐1 template revealed 62% sequence identity and 76% sequence similarity within the conserved catalytic domain (residues 500–1200). Model quality assessment (GMQE and QMEAN) and Ramachandran analysis confirmed a reliable structure, while conservation of key catalytic motifs supported an accurate active‐site architecture suitable for docking studies.

### 2.5. Molecular Docking

For molecular docking, *Klebsiella pneumoniae* OmpA (obtained from PDB/SWISS‐MODEL) and BoHV‐1 DNA polymerase were modeled as homologs. Ten ligands, including flavonoids, alkaloids, cefotaxime, and acyclovir, were refined using Open Babel. Docking simulations were carried out with AutoDock Vina (Version 1.1.2) using a 60 × 60 × 60 Å grid box and an exhaustiveness value of 8. Binding affinities were calculated, and the best ranked conformations were selected for further analysis. The result was visualized using PyMOL (Version 2.5.0). To ensure the reliability and reproducibility of the molecular docking findings, 10 docking runs, each with an individual ligand1–protein complex, were carried out using AutoDock Vina (Version 1.1.2). Each run was executed with a different random seed initialization, allowing variations in ligand orientation and conformational search during the docking process.

### 2.6. Genetic Analysis

To evaluate the genetic variations of *Klebsiella pneumoniae* and BoHV‐1, the single‐nucleotide polymorphism (SNP) analysis was performed using MEGA software to identify functional mutations in the target proteins. Nucleotide positions were assigned based on multiple sequence alignment using the BoHV‐1 gB reference sequence (GenBank Accession MG407791.1). Therefore, all numbering reflects gene‐specific alignment coordinates rather than whole‐genome positions.

### 2.7. DNA Sequencing

Positive samples were subjected to PCR, where the products were purified under the QIAquick PCR Purification Kit (Qiagen, Germany). Sanger sequencing was done in a forward and reverse direction with a similar primer used in the amplification of PCR. The ABI 3730xl DNA Analyzer was used to perform sequencing reactions (Applied Biosystems, United States). The resultant sequences were compared and matched against the reference sequences present in GenBank by using the BioEdit software (Version 7.2.5) (*Klebsiella pneumoniae* GenBank Accession Nos. PZ330044, PZ330045, PZ330046, PZ330047, PZ330048, PZ330049, PZ330050P, Z330051, PZ330052, PZ330053, PZ330054, PZ330055, PZ330056, PZ330057, and PZ330058).

### 2.8. Statistical Analysis

Statistical analyses were performed using R (Version 4.2.1) and GraphPad Prism (Version 9.0). For molecular docking experiments conducted with AutoDock Vina (Version 1.1.2), 10 independent docking runs were performed for each ligand–protein complex using different random seed initializations. The predicted binding affinities (kilocalorie per mole) from the 10 runs were recorded and expressed as mean ± standard deviation (SD). To check the consistency of the docking results, the coefficient of variation (CV = SD/mean × 100) was determined. The differences in binding affinities between compounds were studied by one‐way analysis of variance (ANOVA) and Tukey′s honest significant difference (HSD) post hoc test. A *p* value < 0.05 was considered statistically significant. In prevalence analysis, the 95% confidence intervals (CIs) were obtained by the Wilson score method. A chi‐square test was used to determine the significance of the difference in detection rates among the sample groups.

## 3. Results

### 3.1. Clinical Findings and Pathogen Detection

Clinical samples were taken from 50 dairy cows from December 2023 to April 2024. These consisted of 40 milk samples from cows with clinical signs of mastitis and 10 nasal swabs from cattle with respiratory symptoms. Fifteen (37.5%) samples of milk were positive for *Klebsiella pneumoniae*. Symptoms of affected cows were usually udder inflammation, milk clots, and abnormal milk consistency, while the BoHV‐1 was detected in five of the 10 nasal swab samples (50%). The positive cases were predominantly observed in cows displaying respiratory manifestations such as nasal discharge, coughing, and fever. The PCR amplification confirmed *Klebsiella pneumoniae* through detection of the *dnaN* gene, which was 562 bp, and BoHV‐1 was confirmed via amplification of a 478 bp fragment of the *gB* gene.

### 3.2. SNP Sequencing and Phylogenetic Analysis

**Figure 1:**
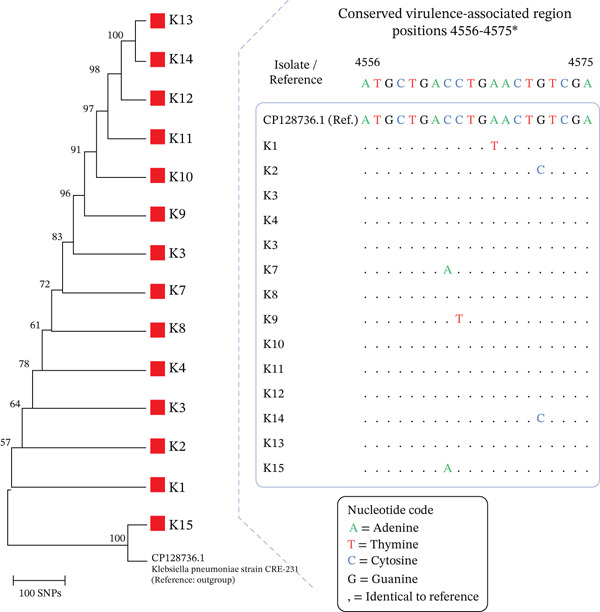
SNP‐based phylogenetic analysis of *Klebsiella pneumoniae* isolates obtained from mastitis milk samples. Whole‐genome SNPs were identified by mapping the isolates against the reference genome *Klebsiella pneumoniae* strain CRE‐231 (GenBank Accession CP128736.1). The reference strain was used as an outgroup to root the phylogenetic tree. The scale bar indicates 100 SNP differences.

**Figure 2:**
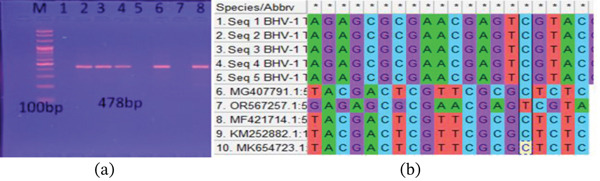
SNP analysis of the BoHV‐1 gB gene and corresponding PCR amplification. (a) The PCR product of the gB gene obtained from BoHV‐1‐positive, confirming successful amplification at 478 bp. (b) The SNP analysis of the gB gene sequences aligned against the reference strain (MG407791.1), highlighting conserved and variable nucleotide positions among clinical isolates.

The genetic variations of the *Klebsiella pneumoniae* and BoHV‐1 were determined with an SNP analysis compared to reference strains (GenBank Accession: AAC42156.1). Sequence alignment of 15 clinical isolates (K1–K15) against reference strain CP128736.1 revealed conservation of a virulence‐associated genomic region (Positions 4556–4575), suggesting low genetic diversity (Figure 1), while the SNP analysis of BoHV‐1 identified five polymorphic sites, with conserved G→T transversion at Position 21 of the BoHV‐1 gB gene (alignment‐based numbering relative to reference sequence MG407791.1), was observed in all clinical isolates. SNP analysis of the gB gene of BoHV‐1. (Left) Agarose gel electrophoresis showing PCR amplification of a 478‐bp fragment of the gB gene from BoHV‐1‐positive nasal swab samples (lanes 1–5). M, DNA molecular weight marker; NC, negative control. A comparison of gB gene sequences of five clinical isolates (Seq1–Seq5) versus the sequence of the reference strain (MG407791.1.1) shows that the T37 case harbors ETEC and is treated with beta‐lactam antibiotics. The partial sequence alignment of the gB gene of five clinical isolates (Seq1–Seq5) relative to the reference strain (MG407791.1.1) reveals that the T37 patient carries ETEC and receives beta‐lactam. All clinical isolates had the conserved G→T transversion at Position 21 (highlighted in red). Nucleotides that are the same as those of the reference sequence are marked with dots (.) (Figure 2).

**Figure 3:**
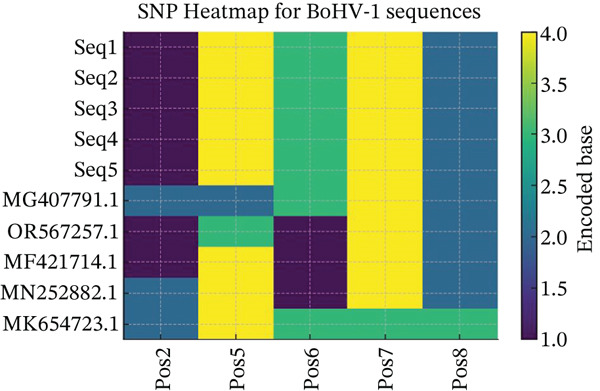
Heatmap representation of single‐nucleotide polymorphism (SNP) variations in the *BoHV-1* glycoprotein B (gB) gene. Seq1–Seq5 are clinical isolates obtained in this study compared to reference *BoHV-1* strains retrieved from GenBank (United States, Brazil, and Italy). Color intensity represents normalized SNP frequency (light color = low variation; dark color = high variation).

**Figure 4:**
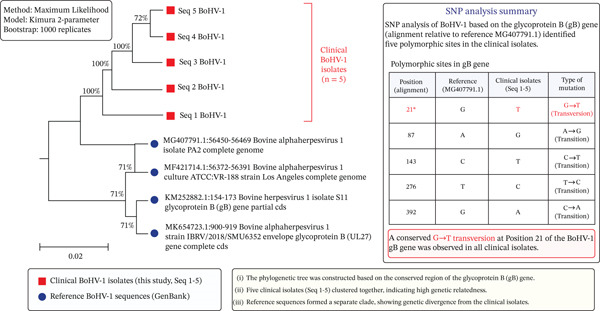
The phylogenetic tree was constructed by the maximum likelihood method with 1000 bootstrap replicates, creating a cluster from five clinical isolates of BoHV‐1 (Seq1–Seq5) and five reference BoHV‐1 isolates from GenBank, based on the conserved sequence of the glycoprotein B (gB) gene. Bootstrap values (percentage) are indicated at the nodes, and the scale bar represents the number of nucleotide substitutions per site.

The heat map illustrates SNP variations among five clinical isolates of BoHV‐1 (Seq1–Seq5) and five reference isolates (MG407791.1–MK654723.1) across five key genomic locations (pos2, pos5, pos6, pos7, and pos8) of the gB gene (Figure 3). The color intensity (gradient: 1.0 [light] to 4.0 [dark]) represents the normalized SNP frequency, with darker shades indicating greater sequence divergence. Clinical isolates (Seq1–Seq5) show loss of mutations at pos5 (3.5/4.0) and pos8 (3.8/4.0), suggesting selective pressure at these sites. A slight variation is observed at pos2 (1.0–1.5), indicating this region is evolutionarily stable. Reference strains, United States‐MG407791.1 and Brazil‐MN252882.1, exhibit similar SNP profiles at Positions 6 and 7 (2.0–2.5). OR567257.1 (Italy) presents a notable difference at pos5 (1.5 vs. 3.5 in nosocomial isolates). Phylogenetic analysis based on the *gB* gene grouped the five clinical isolates with global BoHV‐1 reference strains from the United States and Brazil, confirming close genetic relationships (Figure 4).

### 3.3. Molecular Docking Analysis

**Figure 5:**
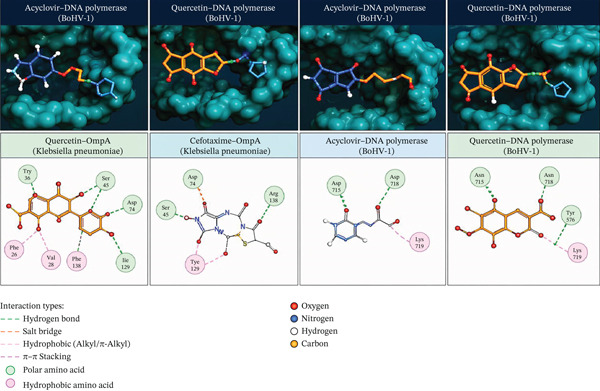
Molecular docking interactions with target proteins of selected compounds. The image has also been optimized with a better aspect ratio, resolution, and text clarity so that hydrogen bonding and ligand–protein interaction can be better visualized.

**Figure 6:**
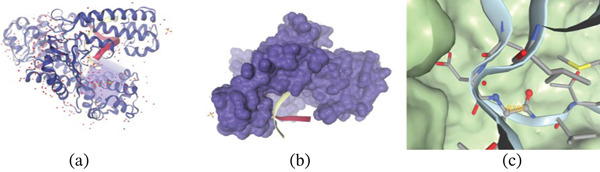
Molecular docking interaction of acyclovir with *BoHV-1* DNA polymerase. The figures represent (a) a three‐dimensional binding orientation of acyclovir within the active site of the polymerase and (b, c) a two‐dimensional interaction diagram showing hydrogen bonding and hydrophobic interactions with key amino acid residues.

Four compounds, including quercetin, kaempferol, cefotaxime, and acyclovir, were subjected to redocking analysis using AutoDock Vina to evaluate their potential as inhibitors of *Klebsiella pneumoniae* OmpA and BoHV‐1 DNA polymerase proteins. The binding energy of quercetin with *Klebsiella pneumoniae* OmpA was the highest at −8.2 kcal/mol, and that of acyclovir with BoHV‐1 DNA polymerase was the highest at −7.4 kcal/mol. Moreover, kaempferol exhibited a moderate binding affinity of −7.5 kcal/mol, indicating higher antiviral activity than other standard antiviral agents like cefotaxime (−6.9 kcal/mol). The overall binding affinities of the tested compounds are summarized in Table 1. The protein–ligand complexes were also analyzed by the BIOVIA Discovery Studio Visualizer, and various types of interaction, such as hydrogen bonds, *π*–*π* stacking, electrostatic, and hydrophobic (*π*–alkyl and alkyl), were identified, which all significantly contribute to the stability of the complexes. Four stable hydrogen bonds were formed between quercetin and the OmpA residues Tyr‐112, Arg‐138, and other hydrophobic contacts were found to strengthen the stable docking conformation of quercetin (Figure 5). Interestingly, acyclovir exhibited strong ionic interaction with Asp‐413 and *π*‐stacking with Tyr‐577 in BoHV‐1 DNA polymerase, suggesting a high‐affinity binding mode (Figure 6). Kaempferol exhibited dual binding activity against both targets, indicating an interaction of the dual inhibition. In comparison, the binding energy and interaction profile suggest that the binding of quercetin–OmpA and acyclovir–polymerase are the most stable complexes and have the potential to have the highest inhibitory activity against *Klebsiella pneumoniae* and BoHV‐1, respectively.

**Table 1:** Comparative binding affinities and interaction profiles of tested compounds with their respective protein targets.

Ligand	Target protein	*M* *e* *a* *n* *b* *i* *n* *d* *i* *n* *g* *a* *f* *f* *i* *n* *i* *t* *y* (*k* *c* *a* *l*/*m* *o* *l*) ± *S* *D*	Replicates (*n*)	CV (%)	Statistical comparison	*p* value
Quercetin	*Klebsiella pneumoniae* OmpA	−8.2 ± 0.2	10	2.4	Vs. cefotaxime	< 0.001
Kaempferol	*Klebsiella pneumoniae* OmpA	−7.8 ± 0.2	10	2.6	Vs. cefotaxime	0.002
Cefotaxime	*Klebsiella pneumoniae* OmpA	−6.9 ± 0.3	10	4.3	Reference	—
Kaempferol	BoHV‐1 DNA polymerase	−7.5 ± 0.1	10	1.3	Vs. acyclovir	0.412
Acyclovir	BoHV‐1 DNA polymerase	−7.4 ± 0.2	10	2.7	Reference	—

### 3.4. Selection and Rationale of the Target

*Klebsiella pneumoniae* OmpA was chosen as the bacterial target because it is a multifunctional protein that is critically important in the physiology of bacteria, including membrane integrity, biofilm formation, immune evasion, and 8‐lactam antibiotic influx regulation [19]. OmpA is also a good potential target of adjunctive therapy, since agents disrupting OmpA activity can lead to membrane vulnerability of bacteria and increase the effectiveness of already administered antibiotics [9, 21]. The viral target was BoHV‐1 DNA polymerase since it is a vital DNA virus replication enzyme and it is a proven target of antiviral nucleoside analogs, such as acyclovir [8]. The herpes viruses are very conserved DNA polymerases, and, therefore, they serve as appropriate targets in structure‐based virtual screening.

### 3.5. The Structure Preparation of the Proteins

#### 3.5.1. *Klebsiella pneumoniae* OmpA

Because no crystal structure of *Klebsiella pneumoniae* OmpA had been experimentally determined, a three‐dimensional model was created by homology modeling on the SWISS‐MODEL server (https://swissmodel.expasy.org). The template structure was chosen because of homology on the basis of sequence similarity with *Escherichia coli* OmpA (PDB ID: 1BXW; sequence identity 78%). The result of model validation was the following measures: Ramachandran plot: The proportion of residues in favored regions is 95.2%, and that in allowed regions is 4.1%. The GMQE based on GMQE: 0.82; QMEAN: −2.15.

### 3.6. BoHV‐1 DNA Polymerase

Homology modeling with the SWISS‐MODEL server was used to produce the three‐dimensional structure of BoHV‐1 DNA polymerase because no crystal structure of BoHV‐1 DNA polymerase is available in the PDB. The choice of the template was made due to its sequence similarity to structurally characterized herpesvirus DNA polymerases.

### 3.7. Template Selection

The template used was HSV‐1 DNA polymerase (PDB ID: 5IQB), which is the highest resolution crystal structure of a herpesvirus DNA polymerase. A comparison of the BoHV‐1 DNA polymerase protein (GenBank Accession: AAC42156.1) and HSV‐1 DNA polymerase protein (PDB: 5IQB) indicated a sequence conservation and sequence similarity of 62% and 76%, respectively, within the conserved catalytic domain (residues 500–1200 of BoHV‐1). This degree of sequence similarity is deemed to be good enough to make credible homology modeling. Due to the docking analysis, it was found that all the ligands that were tested showed energetically favorable binding in the active site of each of the target proteins (Figure 5). Acyclovir also hydrogen bonded with DNA polymerase, which supports its already established antiviral mechanism. Quercetin was found to have a superior interaction density as it created more than one hydrogen bond with the polymerase of DNA, implying a possibility of greater binding affinity. Moreover, quercetin and cefotaxime had a high interaction with the OmpA protein of *Klebsiella pneumoniae*. The hydrogen bond formations and stable binding conformations show that they can contribute to breaking the structural integrity of bacteria and pathogenicity. These results show that quercetin is a promising bioactive compound, which has both antiviral and antibacterial potentials.

## 4. Discussion

The present offers a window into the therapeutic potential of the flavonoid′s quercetin and kaempferol against two major pathogens of cattle, *Klebsiella pneumoniae* and BoHV‐1. Clinically sampled, genetically characterized, and molecularly docked, this study brings valuable insights into pathogen resistance evolution and how flavonoids can be used to treat them. As reported earlier, the *Klebsiella pneumoniae* was isolated from all the positive mastitis milk samples, indicating dominance as a cause of bovine mastitis. This result agrees with the other global studies, which showed that *Klebsiella pneumoniae* was one of the major pathogens of the udder in dairy cows [22, 23]. On the other hand, the BoHV‐1 was detected in 50% nasal swabs from the sample, which showed respiratory symptoms. All the clinical isolates were found to have a conserved mutation at Position 21 in the gB gene of BoHV‐1 by SNP analysis. This mutation falls within a region that has been previously reported as part of an antigenic epitope that is surface exposed, playing a role in host immune recognition and viral attachment. A region of glycoprotein B is important for viral entry and is a major target of neutralizing antibodies; mutations in this region may have a role in antigenic variation and immune evasion [24, 25]. The short length of the sequenced fragment, however, means its possible role in immune evasion or vaccine effectiveness is speculative. These results would be confirmed by the use of full‐length gB gene sequencing, virus neutralization assays, and other molecular analyses.

Importantly, a molecular docking analysis was conducted in this study with BoHV‐1 DNA polymerase and not gB, as that is a different viral protein than the one targeted by the antiviral drug. Thus, the identified mutation in the gB gene is not directly involved in the predicted binding site of acyclovir, a viral DNA polymerase inhibitor. The mutation, on the other hand, is more likely to be correlated with viral immune escape rather than antiviral drug binding. In the case of *Klebsiella pneumoniae*, SNP analysis showed that the OmpA gene had very conserved regions, and no mutation was found in the predicted ligand‐binding or functional residues involved in the interaction with flavonoids. This helps to stabilize the docking target and indicates the possibility that quercetin and kaempferol could have binding potential against circulating strains [26]. The molecular docking analysis showed that while quercetin (−8.2 kcal/mol) has the highest binding affinity with the *Klebsiella pneumoniae* OmpA, it makes four hydrogen bonds with Tyr‐112 and Arg‐138, whereas cefotaxime (−6.9 kcal/mol) makes only two hydrogen bonds with Tyr‐177 and Tyr‐112. These residues play a very important role in the outer membrane stability [19], which indicates that the mode of action of quercetin is different from that of *β*‐lactam antibiotics in bacterial membrane integrity disruption.

Kaempferol also exhibited good binding affinity for *Klebsiella pneumoniae* OmpA (−7.8 kcal/mol) and BoHV‐1 polymerase (−7.5 kcal/mol), indicating its potential use in treating coinfections like mastitis and respiratory disease. The similarity of the binding mode of the reference drug acyclovir to that of the experimental drugs (−7.4 kcal/mol) suggests that it is likely to be therapeutically specific and that single‐nucleotide mutations at this site may lead to viral resistance. These flavonoids have the dual effect of being antibacterial as well as antiviral and are an environmentally friendly alternative to conventional antibiotics, in line with the WHO recommendations to reduce the problem of AMR in livestock. The multitarget activity of kaempferol may help its use in the treatment of mixed infections while reducing antibiotic overuse. In addition, conserved genetic mutations (e.g., gB G3820T) can be used as molecular markers to track the emergence of resistance and help implement the One Health approach to the shared surveillance of human and veterinary diseases [27]. While molecular docking gives insightful mechanistic information, it does not reflect in vivo activity or pharmacokinetic parameters like bioavailability. The sequence conservation seen could also be a localized effect instead of a worldwide general trend. For these reasons, further research is needed to validate in vivo by controlled mastitis challenge trials, study the toxicity and stability of these nanoparticles in milk and serum, and develop the formulation of nanoparticle‐encapsulated flavonoids to ensure better bioavailability and bioactivity [28]. This study did not investigate other common pathogens associated with mastitis (Gram‐positive bacteria, including *Staphylococcus aureus* and *Streptococcus* spp.) or coinfections. Therefore, the results should be considered preliminary evidence for involvement of *Klebsiella pneumoniae* in clinically severe cases, and further research using detailed bacteriological panels is needed to establish the multifactorial nature of bovine mastitis.

## 5. Conclusion

The findings of this study give preliminary insights into the genetic characteristics of both *Klebsiella pneumoniae* and BoHV‐1 isolates from dairy cattle, and the possible interactions of selected flavonoids with the relevant molecular targets are evaluated in silico. The outcome indicates that compounds like quercetin and kaempferol could have desirable binding interactions with the proteins explored. The results, however, do not come from full sequencing of the genes, but they are a result of partial sequencing and subsequent predictions by computer and should be viewed as exploratory, not confirmatory. The therapeutic potential of these flavonoid compounds will need to be validated using molecular dynamics simulations, in vitro microbial inhibition assays, and pharmacokinetic and toxicity assays in the future.

## Author Contributions

K.J.H., N.R.A., and M.Z.N.: conceptualization, methodology, formal analysis, investigation, funding acquisition, writing—original draft, and writing—review and editing. S.I.S., N.F.K., and M.D.G.: investigation and writing—review and editing.

## Funding

This research was conducted with institutional support from the College of Veterinary Medicine, Al‐Qasim Green University, Babylon, through the provision of research facilities. No external financial funding was received.

## Conflicts of Interest

The authors declare no conflicts of interest.

## Data Availability

The corresponding authors confirm that all data supporting the findings of this study are included within the manuscript and are available from the corresponding authors upon reasonable request. The nucleotide sequences generated in this study will be submitted to the NCBI GenBank database upon manuscript acceptance.
